# Development and validation of a nomogram using interpretable machine learning to integrate CT radiomics and PET metabolic parameters for predicting benign-malignant differentiation of pulmonary space-occupying lesions

**DOI:** 10.3389/fradi.2026.1782678

**Published:** 2026-04-21

**Authors:** Xue Liu, Xinghua Liu, Li Bin, Yu Zhang, Huiting Liu, Cailiang Gao

**Affiliations:** 1 Department of Nuclear Medicine, Chongqing University Three Gorges Hospital, Wanzhou, Chongqing, China; 2 Department of Radiology, Chongqing University Three Gorges Hospital, Wanzhou, Chongqing, China

**Keywords:** benign-malignant differential diagnosis, computed tomography, machine learning, nomogram, positron emission tomography, pulmonary space-occupying lesions, radiomics

## Abstract

**Objective:**

To construct a multimodal machine learning model integrating computed tomography (CT) radiomics, Positron Emission Tomography (PET) metabolic parameters, and clinical data for differentiating benign from malignant pulmonary space-occupying lesions (PSOLs), and develop an interpretable nomogram for clinical application.

**Methodology:**

This study enrolled 384 patients with PSOLs who underwent dual-time-point ^1^⁸F-FDG PET/CT examinations. The cohort was divided into a training set (*n* = 268, 145 malignant, 123 benign) and an independent temporal validation set (*n* = 116, 69 malignant, 47 benign) at a 7:3 ratio according to the chronological order of patient enrollment, to avoid data leakage and rigorously assess model generalizability. All malignant lesions were confirmed by pathological examination, while benign lesions were confirmed by pathology (82%) or clinical-imaging follow-up for at least 12 months (18%). CT radiomic features with Intraclass Correlation Coefficient (ICC) values >0.75 were selected, and a Radiomics-score (Rad-score) was generated using the Least Absolute Shrinkage and Selection Operator (LASSO) regression algorithm. Integrated models [Logistic regression, random forest (RF), support vector machine (SVM), eXtreme Gradient Boosting (XGBoost)] were developed by fusing the Rad-score, clinical variables, and PET metabolic parameters. Model performance was evaluated using the area under the receiver operating characteristic curve (AUC), accuracy, sensitivity, specificity, F1-score, and Brier score. Model calibration was assessed via calibration curves, and clinical utility was validated by decision curve analysis (DCA). Model interpretability was achieved using SHapley Additive exPlanations (SHAP) values for the optimal XGBoost model, and a clinically applicable, interpretable nomogram was constructed based on the core predictive features identified by SHAP analysis to facilitate clinical translation.

**Results:**

A Rad-score was constructed from 17 optimally selected features. In the independent temporal validation set, the single-modality models achieved AUCs of 0.808 (Radiomics Model), 0.732 (Clinical Model), and 0.874 (Metabolic Model). Among all tested models, the XGBoost integrated model achieved the highest AUC of 0.967, which was significantly higher than that of all other models (Bonferroni-adjusted *P* = 0.002–0.032, all adjusted *P* < 0.05). SHAP analysis identified *Δ*SUVmax, Rad-score, and delayed phase total lesion glycolysis (TLG_d) as the top three key predictive features.

**Conclusions:**

The predictive logic of the optimal XGBoost model was decoded via SHAP analysis to identify core predictive features, and a clinically applicable, interpretable nomogram was further established based on a multivariate logistic regression model using these core features, to facilitate the clinical translation of our model.

## 1 Introduction

Pulmonary space-occupying lesions (PSOLs) are focal abnormalities frequently encountered in imaging studies and carry significant clinical implications, as they may represent a spectrum of conditions including lung cancer, benign tumors, and infectious processes (1). Accurate characterization is crucial for guiding treatment and prognostic assessment. The current diagnostic paradigm relies on computed tomography (CT), positron emission tomography/computed tomography (PET/CT), and pathological examination. However, these methods have limitations: CT interpretation is highly dependent on radiologists’ expertise, with inter-observer diagnostic variability reaching 15%–20% (2); PET/CT has suboptimal specificity, as approximately 30% of benign lesions (e.g., tuberculosis) can exhibit high metabolic uptake mimicking malignancy (3); invasive biopsies carry risks such as bleeding and pneumothorax, with lower success rates for deep-seated or small lesions (4). Consequently, there is a pressing need for accurate non-invasive diagnostic methods.

In recent years, predictive models have opened new avenues for diagnostic approaches (5). By integrating multi-source heterogeneous data—such as radiomic features, PET metabolic parameters, and clinical indicators—these models can uncover complex patterns and enhance diagnostic performance (6). In pulmonary diseases, the combination of CT radiomics with PET parameters has shown significant promise (7). For example, studies have reported that integrated models can improve diagnostic accuracy, e.g., from 67% to 75%, compared with single-source models (8). Kang et al. (9), in a study of 268 patients, demonstrated that a PET/CT radiomics model reduced the false-positive rate from 30.6% to 5.4%, achieving an AUC of 0.89. Nevertheless, most existing studies have focused on single modalities or pairwise combinations, with limited research exploring the comprehensive integration of three data modalities: CT radiomics, dual-time-point PET metabolic parameters, and clinical data.

Machine learning offers a pivotal solution to this challenge of multi-modal data integration (10). Its algorithms inherently support the handling of high-dimensional, heterogeneous data and can capture nonlinear relationships across different data sources, thereby providing a robust technical framework for the comprehensive fusion of CT radiomic features, PET metabolic parameters, and clinical information (11). Unlike conventional statistical methods, which are often constrained by linear assumptions and simplified data processing, machine learning models—including logistic regression, random forest (RF), support vector machine (SVM), and eXtreme Gradient Boosting (XGBoost)—can effectively capture optimal feature fusion strategies and effectively extract valuable diagnostic information hidden within complex multi-source interactions (12).

Therefore, this study aims to integrate CT radiomic features, PET metabolic parameters, and clinical data from patients with pulmonary space-occupying lesions, in order to construct and compare multiple machine learning models. SHAP analysis will be applied to the optimal model to identify key predictive features, and a clinically interpretable nomogram will be developed to assist physicians in accurately distinguishing between benign and malignant pulmonary lesions.

## 2 Materials and methods

### 2.1 Study design

This single-center retrospective study was performed in full accordance with the Declaration of Helsinki and institutional ethical requirements. The core study protocol was approved by the Ethics Committee of Chongqing University Three Gorges Hospital prior to patient enrollment (Initial Approval No. 2022-KY-088, June 2022), with supplementary review and re-approval of the full study workflow completed by the same committee on 27 June 2025 (Supplementary Approval No. 2022-KY-088-1). Written informed consent was waived given the retrospective, fully anonymized nature of the study.

The final diagnosis of all included PSOLs was confirmed with a strict standard: all malignant lesions were definitively diagnosed by pathological examination (surgical resection or needle biopsy); 82% of benign lesions were confirmed by pathology, and the remaining 18% of benign lesions were confirmed by clinical and imaging follow-up for at least 12 months with no signs of progression.

The cohort was divided into a training set (the first 70% of patients enrolled chronologically, *n* = 268) and an independent temporal validation set (the remaining 30% of patients, *n* = 116) in a 7:3 ratio. This temporal split strategy was adopted to completely avoid data leakage, as the validation set was fully isolated from all steps of feature selection, hyperparameter tuning, and model training, which can more rigorously assess the model's real-world generalizability than random split. The exact benign-malignant breakdown was: training set (145 malignant, 123 benign), validation set (69 malignant, 47 benign).

### 2.2 Study population

Inclusion criteria were: (1) Patients with pulmonary space-occupying lesions confirmed by pathology or clinical follow-up; (2) Completion of an ^1^⁸F-FDG PET/CT examination with complete imaging data and no prior antitumor therapy directed at the pulmonary lesion before the scan; (3) Availability of complete clinical data.

Exclusion criteria were: (1) Undergoing PET/CT after initiation of antitumor therapy; (2) Severe image artifacts or unmeasurable metabolic parameters; (3) Missing >20% of clinical or laboratory data.

### 2.3 Image data acquisition and preprocessing

All PET/CT examinations were performed on a single United Imaging uMI 780 PET/CT scanner (United Imaging Healthcare, Shanghai, China), which integrates a 128-slice helical CT system and a time-of-flight (TOF) PET system. The core technical parameters of the system are as follows: (1) PET system: LYSO scintillation crystal, axial field of view (FOV) 26.3 cm, transaxial FOV 70 cm, spatial resolution 3.8 mm at 1 cm from the center of FOV, time resolution 390 ps, sensitivity 18 cps/kBq at the center of FOV; (2) CT system: 128-slice detector configuration, spatial resolution 15 lp/cm at the center of FOV, tube voltage range 80–140 kV, maximum tube current 600 mA, rotation time 0.5 s.

Completely consistent acquisition and reconstruction protocols were used for all enrolled patients throughout the entire study period (January 2023 to June 2025), with no changes in equipment parameters, operators, or scanning workflow, to minimize inter-scan variability and avoid batch effects. This design fully complies with the Image Biomarker Standardization Initiative (IBSI) guidelines for reproducible radiomic research. No ComBat or other batch effect correction algorithm was applied in this study, as all data were acquired from the same scanner with uniform imaging parameters, with no significant inter-scan batch effect observed.

All patients fasted for at least 6 h prior to the examination, ensuring blood glucose levels were controlled at <11.1 mmol/L. ^1^⁸F-FDG was administered intravenously at a dose of 3.7–5.5 MBq/kg body weight after passing quality control. Early imaging was conducted 40–60 min post-injection, and delayed imaging was performed approximately 2 h (120 ± 10 min) post-injection, both covering from the skull vertex to the mid-femur.

CT scanning parameters: tube voltage 120 kV, automatic tube current modulation, slice thickness 3 mm, slice increment 3 mm, pitch 0.984, matrix 512 × 512.

PET reconstruction parameters: PET emission data were acquired in 3D mode, reconstructed using the Ordered Subset Expectation Maximization (OSEM) algorithm (2 iterations, 21 subsets) with CT-based attenuation correction, matrix 256 × 256, pixel size 2.73 × 2.73 mm.

The reconstructed PET and CT images were fused to generate axial, coronal, and sagittal images for subsequent lesion segmentation and parameter extraction.

### 2.4 Radiomic feature extraction and selection

#### 2.4.1 Lesion segmentation

CT images of all patients were imported into 3D Slicer software (version 4.13, https://www.slicer.org) for lesion segmentation. Two attending physicians with 5 and 7 years of experience in thoracic imaging diagnosis, respectively, completed the segmentation under a strict double-blinded design: both physicians were blinded to the patients’ pathological results (benign/malignant class of the lesion), clinical information, PET metabolic parameters, and each other's segmentation results during the entire process.

Before formal segmentation, the two physicians completed standardized training and reached a full consensus on the lesion delineation rules: for each lesion, the volume of interest was manually delineated layer by layer to fully cover the entire tumor parenchyma, excluding the surrounding normal lung tissue, bronchi, and blood vessels, rather than only delineating a single slice. These rules were strictly followed for all lesions, regardless of the lesion's size, morphology, or suspected benign/malignant nature.

The final segmentation workflow for all 384 lesions was as follows: the initial segmentation was completed by Physician A, and then independently verified by Physician B; any discrepancies in segmentation were resolved through consensus discussion between the two physicians, and the final segmentation result for feature extraction was confirmed by both. Notably, the two physicians did not divide the segmentation work according to the lesion class, and both completed segmentation and verification for both benign and malignant lesions, which completely avoided artificial differences between groups caused by different radiologists segmenting different classes of lesions.

#### 2.4.2 Segmentation reproducibility assessment

To evaluate the stability of radiomic features, 30 cases were randomly selected from the training set for reproducibility testing, with no access to the validation set data to avoid data leakage. The sample size of 30 cases is fully compliant with the recommendation of the Radiomics Quality Score guidelines, which require a minimum of 20–30 cases for inter-observer and intra-observer reproducibility validation of radiomic features (13). For ICC calculation with a predefined stability threshold of ICC > 0.75, this sample size achieves a statistical power of >90%, which is sufficient to accurately distinguish stable features from unstable ones.

The 30 selected cases included a 1:1 balanced distribution of malignant (*n* = 15) and benign (*n* = 15) lesions, which exactly matched the class distribution of the overall cohort, to completely avoid class imbalance bias in reproducibility assessment. Meanwhile, the 30 cases covered the full spectrum of lesion size, location, and morphological types in the overall cohort, to ensure the representativeness of the assessment results.

The reproducibility assessment workflow was as follows: (1) Intra-observer reproducibility: Physician A repeated the segmentation of the 30 lesions 1 month after the initial segmentation, with no access to the previous segmentation results; (2) Inter-observer reproducibility: Physician B independently completed the segmentation of the 30 lesions, with no access to Physician A's segmentation results; (3) ICC calculation: The intraclass correlation coefficient was calculated using a two-way mixed-effects model, absolute agreement definition, single measures (the standard model for radiomic feature reproducibility assessment). Only features with both intra-observer ICC > 0.75 and inter-observer ICC > 0.75 were retained as stable features for subsequent analysis. The ICC values of the retained stable features ranged from 0.752 to 0.986, with a median ICC of 0.891, indicating excellent stability of the selected features.

#### 2.4.3 Radiomic feature extraction

A total of 851 radiomic features, fully compliant with the Image Biomarker Standardization Initiative (IBSI) guidelines, were extracted from the validated volume of interest (VOI) using the SlicerRadiomics extension (version 3.0.1) in 3D Slicer. The complete feature system is classified as follows:

(1) 14 shape features: describing the 3D geometric morphology of the lesion, including Flatness, MajorAxisLength, Sphericity, VoxelVolume, etc., to quantify the macroscopic shape and border characteristics of the lesion; (2) 18 first-order statistical features: reflecting the distribution of CT attenuation values within the lesion, including 10Percentile, 90Percentile, Kurtosis, Mean, etc., to characterize the overall density level and distribution pattern of the lesion; (3) 819 textural features, including: 24 features from Gray-Level Co-occurrence Matrix (GLCM); 14 features from Gray-Level Dependence Matrix (GLDM); 16 features from Gray-Level Run-Length Matrix (GLRLM); 16 features from Gray-Level Size Zone Matrix (GLSZM); 5 features from Neighboring Gray Tone Difference Matrix (NGTDM); 744 features derived from 6 types of 3D wavelet transformations (LLH, LHL, HLL, LHH, HLH, HHH wavelet filters) of the original CT images. Wavelet transformation decomposes the original image into different frequency domains, to capture fine-grained intratumoral heterogeneity and subtle density/texture changes that cannot be identified in the original image domain.

#### 2.4.4 Feature selection pipeline

##### 2.4.4.1 Core principle of no data leakage

All feature selection steps, Z-score standardization parameter fitting, and LASSO model training were performed strictly and exclusively within the training set only. The independent temporal validation set was completely isolated throughout the entire process, with no access to any feature selection, model training, or parameter fitting steps. The Rad-score of the validation set was calculated using only the standardization parameters and LASSO coefficients obtained from the training set, to completely eliminate data leakage and ensure the reliability of model performance evaluation. Notably, the screening of clinical and metabolic features also strictly followed the same no-data-leakage principle, with all screening steps performed only within the training set.

##### 2.4.4.2 Stepwise radiomic feature selection pipeline

The pipeline was designed for dimensionality reduction of high-dimensional radiomic features, with no redundant operations, and the specific steps were as follows: (1) Stability filtering: Retain only features with both intra-observer ICC > 0.75 and inter-observer ICC > 0.75, to eliminate features with poor segmentation reproducibility; (2) Standardization: Perform Z-score standardization on all retained stable features, with the standardization parameters fitted only on the training set; (3) Collinearity removal: Remove highly collinear features with Spearman correlation coefficient r > 0.9, to avoid feature redundancy and reduce model overfitting risk; (4) Predictive feature screening: Select features with non-zero coefficients via LASSO logistic regression with 10-fold cross-validation (conducted only within the training set), using the *λ*.1se criterion to balance model performance and complexity.

##### 2.4.4.3 Rad-score calculation and model input rules

A Radiomics-score (Rad-score) was calculated for each patient by summing the product of the LASSO-selected radiomic features and their corresponding non-zero coefficients. The Rad-score was treated as the only integrated radiomic predictor in all subsequent models (including the single-modality Radiomics Model and the multi-modal Integrated Model). Raw radiomic features were not re-entered into any machine learning models in this study, to avoid high-dimensional feature redundancy, dimensional explosion, and model overfitting. The screening of clinical and metabolic features was performed via an independent pipeline (univariable+multivariable logistic regression) for low-dimensional variables, with no overlap or redundant operations with the radiomic feature selection process.

### 2.5 Clinical and metabolic feature collection

The following metabolic parameters were extracted from the dual-time-point PET images: delayed phase maximum standardized uptake value (SUVmax_d), early phase maximum standardized uptake value (SUVmax_e), and their percentage change (*Δ*SUVmax); delayed phase metabolic tumor volume (MTV_d), early phase metabolic tumor volume (MTV_e), and their percentage change (*Δ*MTV); delayed phase total lesion glycolysis (TLG_d), early phase total lesion glycolysis (TLG_e), and their percentage change (*Δ*TLG). All percentage changes (*Δ*) were calculated using the formula: [(Delayed phase value - Early phase value)/Early phase value] × 100%. Additionally, patient clinical data were collected, including sex, age, smoking history, and serum tumor marker levels: Pro-gastrin-releasing peptide (ProGRP), neuron-specific enolase (NSE), carcinoembryonic antigen (CEA), and squamous cell carcinoma antigen (SCC), all serum tumor markers were measured within 2 weeks prior to the PET/CT examination.

### 2.6 Predictive model construction and evaluation

The Integrated Model strictly followed the feature input rules defined in the radiomic feature selection pipeline: only the pre-calculated Rad-score, screened clinical predictors, and screened metabolic predictors were used as model inputs, with no raw radiomic features included. This study developed a multi-dimensional predictive model system using a standardized evaluation pipeline. Three single-modality models—Radiomics, Clinical, and Metabolic—were constructed using logistic regression. The Radiomics Model employed LASSO regression (10-fold cross-validation, *λ*.1se criterion) to select radiomic features with non-zero coefficients, forming the Rad-score as its core predictor. The Clinical and Metabolic Models first applied univariable logistic regression (*P* < 0.100) for initial screening of clinical indicators or metabolic parameters, followed by multivariable logistic regression (backward stepwise selection, AIC) to determine the final features.

The Integrated Model adopted a feature-level fusion strategy, combining the pre-screened radiomic signature (Rad-score), clinical variables, and PET metabolic parameters to form a standardized fixed feature set. Four mainstream machine learning algorithms, including Logistic Regression (LR), Random Forest (RF), Support Vector Machine (SVM), and eXtreme Gradient Boosting (XGBoost), were trained on this unified feature set under a strict no-data-leakage hyperparameter optimization pipeline, with all tuning procedures performed exclusively within the training set and no access to the independent temporal validation set throughout the entire process.

For each algorithm, we predefined a hyperparameter grid with a core focus on embedding regularization constraints to limit model complexity and mitigate overfitting risks. Hyperparameter optimization was conducted via 5-fold cross-validation (CV) and grid search: in each fold of the 5-fold CV, the training subset of the corresponding fold was used for model fitting, while the held-out validation subset was applied for performance evaluation, and the hyperparameter combination yielding the highest mean Area Under the Curve (AUC) across the 5 folds was selected as the optimal parameter set. With the finalized optimal hyperparameters, we retrained each model on the full training set to obtain the final predictive model, which was subsequently used for prediction on the completely isolated temporal validation set.

The detailed hyperparameter search space and overfitting-prevention regularization settings for each algorithm were predefined as follows: for Logistic Regression (LR), we set the penalty term as L1 and L2 (to reduce feature redundancy and overfitting), regularization strength C as [0.01, 0.1, 1, 10, 100] (smaller C represents stronger regularization), the optimization metric as AUC, and the solver as liblinear adapted to the small-sample high-dimensional feature set in this study; for Random Forest (RF), the search range included number of decision trees (n_estimators) [100, 200, 300], maximum tree depth (max_depth) [5, 10, 15, None] (restricting tree depth to avoid overfitting to noise features), minimum samples required for node splitting (min_samples_split) [2, 5, 10], minimum samples required for leaf nodes (min_samples_leaf) [1, 2, 4], and number of features considered for splitting (max_features) [“sqrt”, “log2”], with the optimization metric set as AUC and bootstrap sampling enabled for all tree constructions to enhance model robustness; for Support Vector Machine (SVM), we set regularization strength C as [0.01, 0.1, 1, 10, 100], kernel function as [“linear”, “rbf”], kernel coefficient (gamma) as [“scale”, “auto”], the optimization metric as AUC, and class weight as “balanced” to adapt to the slight class imbalance of the cohort.

For the eXtreme Gradient Boosting (XGBoost) model, which was the optimal model in this study, the search space included maximum tree depth (max_depth) [3, 4, 5, 6] (strictly limited to shallow trees to avoid overfitting), learning rate [0.01, 0.05, 0.1], number of boosting rounds (n_estimators) [50, 100, 200], row subsampling ratio (subsample) [0.7, 0.8, 0.9] and column subsampling ratio (colsample_bytree) [0.7, 0.8, 0.9] (both random sampling strategies were applied to reduce overfitting), L1 regularization term (reg_alpha) [0, 0.1, 1], and L2 regularization term (reg_lambda) [0, 0.1, 1]; the optimization metric was AUC, and the random seed was fixed for all iterations to ensure the reproducibility of the results.

To further rigorously evaluate the model’s generalization performance and rule out overfitting bias caused by single 5-fold CV, we additionally performed a 5 × 5 nested cross-validation (NCV) analysis entirely within the training set (no data from the independent temporal validation set was involved in the whole process): (1) Outer loop: 5-fold CV of the training set, used for unbiased performance evaluation of the model; (2) Inner loop: 5-fold CV of the outer training subset, used for hyperparameter grid search and optimal parameter selection; (3) The mean ± standard deviation (SD) of the performance metrics of the nested CV were reported to quantitatively assess the model’s robustness.

All models used a unified classification threshold of 0.5 to distinguish between malignant and benign lesions, to ensure consistent and comparable performance metrics across all models. Model discrimination performance was assessed using AUC (with 95% confidence interval, 95% CI), accuracy, sensitivity, specificity, F1-score, and Brier score. Differences in AUC between models were compared using DeLong's test. Additionally, Net Reclassification Improvement (NRI) and Integrated Discrimination Improvement (IDI) with 95% CIs were calculated to quantify the incremental predictive value of the integrated model over single-modality models.

Model calibration performance was evaluated via calibration curves and the Hosmer-Lemeshow test in both the training and validation sets, to assess the consistency between the model-predicted malignancy probability and the actual observed probability. The clinical utility of the model was validated by decision curve analysis (DCA), which quantified the net benefit of the model under different clinical decision thresholds.

### 2.7 Model interpretability and nomogram construction

Based on the best-performing XGBoost integrated model, SHapley Additive exPlanations (SHAP) values were utilized to decode the model's decision logic, quantify the relative contribution of each feature, and generate global and local interpretability analyses. To facilitate clinical translation, the core predictive features with the highest global importance ranked by SHAP values were selected to construct a multivariate logistic regression model, and a clinically applicable nomogram was further developed based on this logistic regression model using the rms package in R. This nomogram enables intuitive, visual calculation of the individual malignancy risk of PSOLs for clinicians.

### 2.8 Statistical analysis

All statistical analyses were performed using R (version 4.2.0) and Python (version 3.9), with a two-sided *P*-value < 0.05 considered statistically significant. Continuous variables were assessed for normality using the Shapiro–Wilk test; normally distributed data were expressed as mean ± standard deviation and compared using the independent samples t-test, while non-normal data were presented as median (interquartile range) and compared with the Mann–Whitney U test. Categorical variables were described as frequency (percentage) and analyzed using the chi-square test or Fisher's exact test, as appropriate. These methods were used to compare baseline characteristics between training and test sets to evaluate data split balance. Model performance was primarily evaluated using AUC with 95% confidence intervals (95% CI). DeLong's test was applied for pairwise AUC comparisons, and NRI and IDI with 95% CIs were computed to quantitatively assess improvements in risk classification and discriminatory power. Calibration curves were plotted to visualize the agreement between predicted and actual probabilities, with the Hosmer-Lemeshow test used to assess statistical significance of calibration deviation. Decision curve analysis was performed to calculate the net benefit of the model across a range of threshold probabilities, to evaluate its clinical application value.

## 3 Results

### 3.1 Patient baseline characteristics

**Figure 1:**
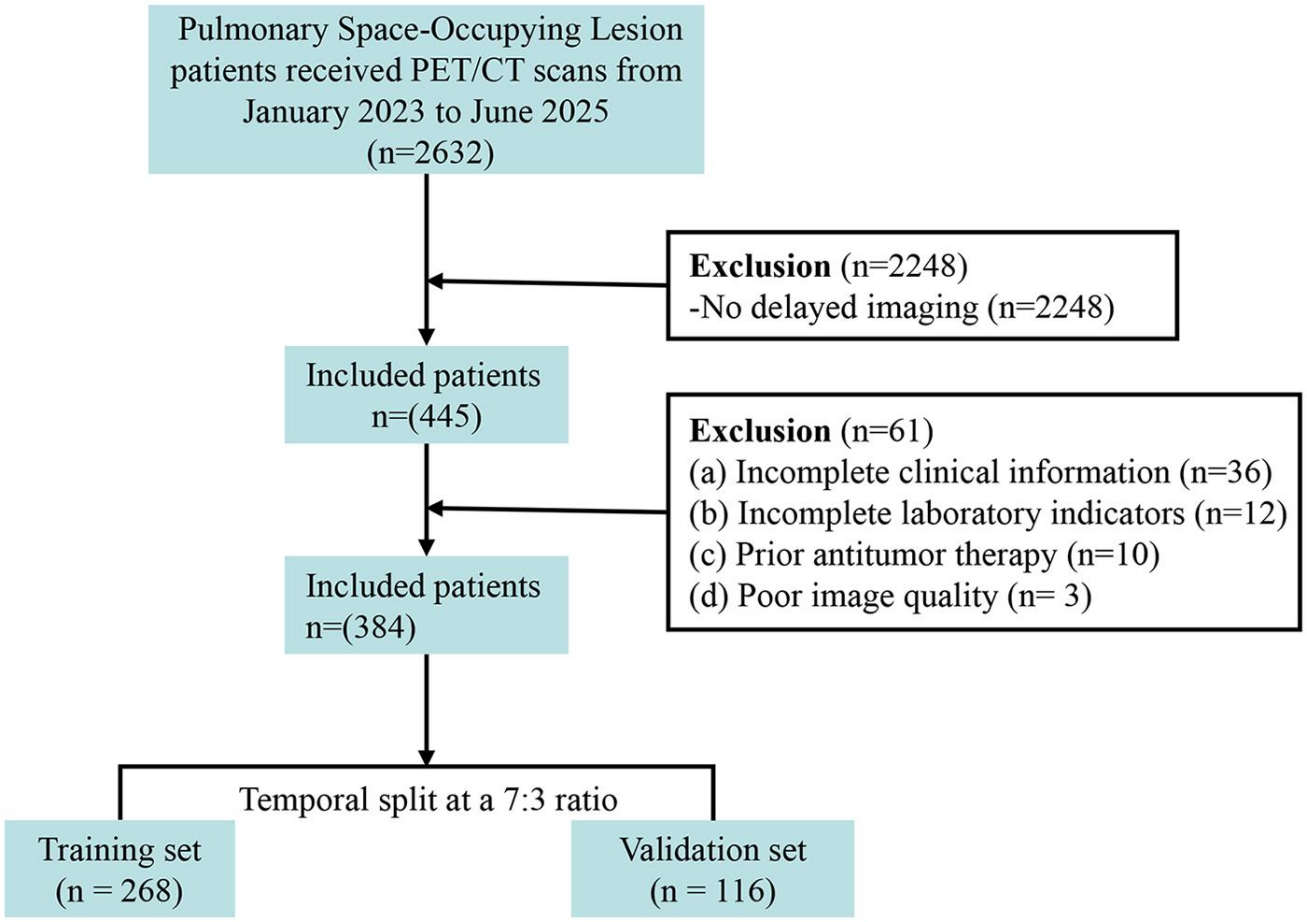
Study flowchart.

Based on the inclusion and exclusion criteria, a total of 384 patients with PSOLs who underwent ^1^⁸F-FDG PET/CT examination were finally enrolled (Figure 1). Among them, 214 (55.7%) patients had malignant lesions (all pathologically confirmed) and 170 (44.3%) had benign lesions (82% pathologically confirmed, 18% confirmed by ≥12 months follow-up). The cohort was split into a training set (268 patients, 145 malignant, 123 benign) and an independent temporal validation set (116 patients, 69 malignant, 47 benign) in a 7:3 ratio based on patient enrollment chronological order, which was consistent with the split strategy described in the study design. The median patient age was 68.68 ± 12.47 years, and 52.1% were male.

Baseline characteristics were well-balanced between the training and independent temporal validation sets (all *P* > 0.05), indicating no significant systematic difference between the two cohorts. Significant differences were observed in multiple clinical and metabolic parameters between the malignant and benign groups (*P* < 0.05), confirming the predictive value of these features for benign-malignant differentiation (Table 1).

**Table 1:** Baseline characteristics of 384 patients with PSOLs undergoing ^1^⁸F-FDG PET/CT.

Characteristic	All (*n* = 384)	Training (*n* = 268)	Validation (*n* = 116)	*P* value (training vs. validation)	Malignant (*n* = 214)	Benign (*n* = 170)	*P* value (malignant vs. benign)
Age, mean ± SD	68.68 ± 12.47	68.58 ± 12.51	68.92 ± 12.43	0.805	71.24 ± 10.35	65.42 ± 13.89	<0.001
Sex, *n* (%)				0.274			0.001
Male	200 (52.1)	145 (54.1)	55 (47.4)		128 (59.8)	72 (42.4)	
Female	184 (47.9)	123 (45.9)	61 (52.6)		86 (40.2)	98 (57.6)	
Smoking, *n* (%)				0.962			<0.001
Yes	140 (36.5)	97 (36.2)	43 (37.1)		102 (47.7)	38 (22.4)	
No	244 (63.5)	171 (63.8)	73 (62.9)		112 (52.3)	132 (77.6)	
SCC, mean ± SD	1.31 ± 0.66	1.30 ± 0.64	1.34 ± 0.71	0.562	1.38 ± 0.71	1.22 ± 0.58	0.021
CEA, mean ± SD	6.66 ± 5.64	6.42 ± 5.49	7.21 ± 5.97	0.213	9.87 ± 6.12	2.65 ± 2.18	<0.001
NSE, mean ± SD	23.94 ± 21.49	23.25 ± 19.71	25.55 ± 25.15	0.336	26.72 ± 24.15	20.45 ± 17.23	0.005
ProGRP, mean ± SD	35.54 ± 2.94	35.45 ± 2.91	35.76 ± 3.01	0.351	35.82 ± 3.05	35.19 ± 2.76	0.038
TLG_d, mean ± SD	144.62 ± 76.80	143.91 ± 77.58	146.27 ± 75.27	0.783	189.54 ± 68.72	88.23 ± 52.41	<0.001
TLG_e, mean ± SD	149.43 ± 80.53	145.93 ± 77.85	157.52 ± 86.22	0.196	182.36 ± 75.43	108.12 ± 72.58	<0.001
*Δ*TLG, mean ± SD	0.33 ± 1.24	0.36 ± 1.25	0.28 ± 1.21	0.596	0.87 ± 1.32	−0.35 ± 0.86	<0.001
SUVmax_e, mean ± SD	10.46 ± 4.40	10.27 ± 4.50	10.89 ± 4.14	0.202	13.25 ± 3.87	6.98 ± 3.02	<0.001
SUVmax_d, mean ± SD	11.86 ± 4.57	11.66 ± 4.71	12.31 ± 4.24	0.203	15.12 ± 3.64	7.78 ± 3.15	<0.001
*Δ*SUVmax, mean ± SD	2.52 ± 16.70	2.22 ± 17.31	3.19 ± 15.23	0.603	12.84 ± 14.26	−10.52 ± 12.37	<0.001
MTV_e, mean ± SD	37.53 ± 20.15	37.18 ± 18.97	38.33 ± 22.70	0.610	45.82 ± 18.64	27.05 ± 16.83	<0.001
MTV_d, mean ± SD	60.72 ± 48.77	61.85 ± 49.48	58.13 ± 47.20	0.494	78.64 ± 45.23	38.22 ± 36.75	<0.001
*Δ*MTV, mean ± SD	1.26 ± 4.05	1.44 ± 4.61	0.86 ± 2.22	0.201	2.15 ± 4.87	0.14 ± 2.12	<0.001

SCC, squamous cell carcinoma antigen; CEA, carcinoembryonic antigen; NSE, neuron-specific enolase; ProGRP, Pro-gastrin-releasing peptide; TLG_d, delayed phase total lesion glycolysis; TLG_e, early phase total lesion glycolysis; *Δ*TLG, percentage change in TLG; SUVmax_e, early phase maximum standardized uptake value; SUVmax_d, delayed phase maximum SUV; *Δ*SUVmax, percentage change in SUVmax; MTV_e, early phase metabolic tumor volume; MTV_d, delayed phase MTV; *Δ*MTV, percentage change in MTV.

### 3.2 Radiomics feature selection

A total of 851 radiomic features were extracted from the 3D VOIs of the 384 patients. Following the reproducibility assessment, 183 features (21.5%) with both intra-observer and inter-observer ICC > 0.75 were retained as high-stability features, of which textural features constituted 82.6%, and wavelet-transformed features accounted for the majority of the retained features. The ICC values of the retained features ranged from 0.752 to 0.986, with a median of 0.891.

**Figure 2:**
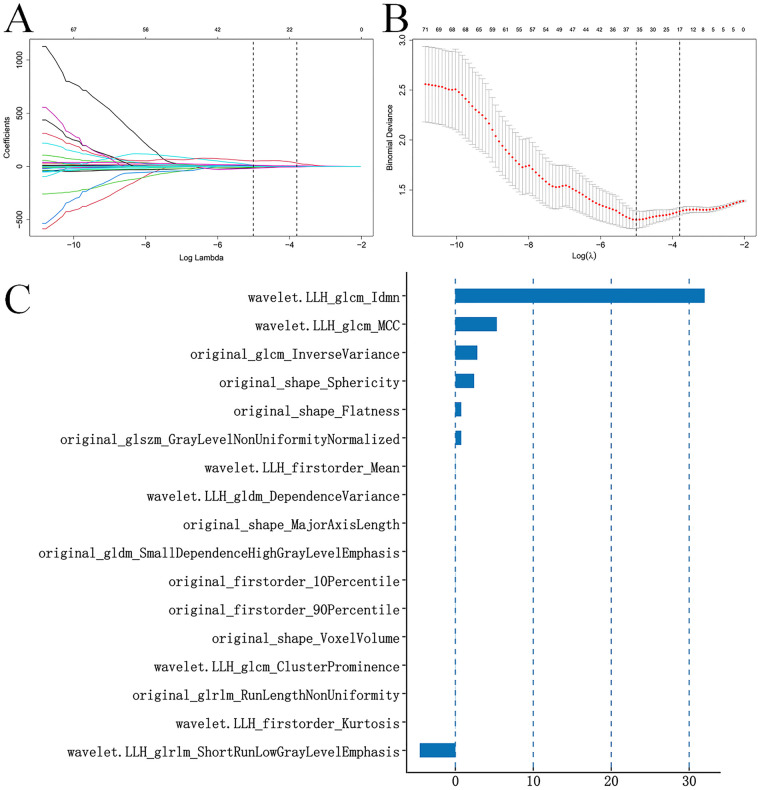
LASSO coefficients of radiomic features. **(A)**: LASSO coefficient profile of 71 radiomic features. A vertical line is generated at the logarithmic (*λ*) scale using ten-fold cross-validation, where the optimal *λ* value (0.022398) was selected to retain 17 radiomic features with non-zero coefficients. The top *X*-axis indicates the count of features with non-zero coefficients in the model. **(B)**: A black vertical line is plotted at the *λ* value selected via ten-fold cross-validation in **(A)** The top *X*-axis denotes the number of features with non-zero coefficients in the model. **(C)**: Bar plot of the non-zero LASSO coefficients for the 17 selected radiomic features. The *Y*-axis lists the 17 radiomic features, and the *X*-axis shows their corresponding LASSO coefficients.

Subsequently, univariate association analysis (all *P* < 0.01 after Benjamini-Hochberg correction) combined with removal of collinear features (Pearson correlation coefficient |r| < 0.9) resulted in 71 non-redundant features (38.8% of the 183 stable features). Finally, 17 features with non-zero coefficients were selected via LASSO regression (10-fold cross-validation within the training set, *λ*.1se criterion) to construct the Radiomics-score (Rad-score) (Figure 2). The full list of the 17 core features, their corresponding LASSO coefficients, ICC values, and between-group difference *P* values are provided in Supplementary Table S1. All 17 selected features showed significant differences between the malignant and benign groups in the training set (all *P* < 0.001).

The Rad-score ranged from −4.97 to 3.25 in the training set (*n* = 268), with the malignant group showing a significantly higher mean score than the benign group (*P* < 0.001). Its range in the independent temporal validation set (*n* = 116) was −2.65 to 2.75, with a consistent significant difference between malignant and benign groups (*P* < 0.001), indicating robust generalization performance of the Rad-score.

The final fixed, fully disclosed calculation formula of Rad-score is as follows:

Rad-score=−37.4693 + 0.7563 × original_shape_Flatness + 0.0056  × original_shape_MajorAxisLength + 2.3994  × original_shape_Sphericity+8.96 × 10^−6^  × original_shape_VoxelVolume + 0.0010  × original_firstorder_10Percentile + 0.000367 × original_firstorder_90Percentile + 2.8237  × original_glcm_InverseVariance + 0.0025  × original_gldm_SmallDependenceHighGrayLevelEmphasis−5.7 × 10^−6^  × original_glrlm_RunLengthNonUniformity + 0.7458  × original_glszm_GrayLevelNonUniformityNormalized−0.0138  × wavelet.LLH_firstorder_Kurtosis + 0.0089  × wavelet.LLH_firstorder_Mean + 1.6 × 10^−6^  × wavelet.LLH_glcm_ClusterProminence + 31.9905  × wavelet.LLH_glcm_Idmn + 5.3096  × wavelet.LLH_glcm_MCC + 0.0080  × wavelet.LLH_gldm_DependenceVariance−4.5512  × wavelet.LLH_glrlm_ShortRunLowGrayLevelEmphasis.

### 3.3 Feature selection and multivariable analysis

Univariable analysis identified sex, age, smoking, CEA, TLG_d, *Δ*SUVmax, MTV_d, *Δ*MTV, *Δ*TLG, and SUVmax_d as significantly associated with malignant lesions (all *P* < 0.100, Table 2). Subsequent multivariable logistic regression (backward stepwise selection, Akaike Information Criterion - AIC) identified nine independent predictors, including clinical features (sex, smoking, CEA) and metabolic features (TLG_d, *Δ*TLG, SUVmax_d, *Δ*SUVmax, MTV_d) (all *P* < 0.05, Table 2).

**Table 2:** Univariate and multivariate analysis of factors predicting lung lesions in the training dataset.

Characteristics	Univariate analysis		Multivariate analysis	
	OR (95% CI)	*P* value	OR (95% CI)	*P* value
Age	1.044 (1.023–1.067)	<0.001	1.041 (1.017–1.066)	<0.001
Sex, Male vs. Female	2.29 (1.402–3.775)	<0.001	2.445 (1.406–4.251)	0.002
ProGRP	1.001 (0.922–1.088)	0.976	-	-
NSE	1.006 (0.993–1.022)	0.429	-	-
CEA	1.117 (1.059–1.187)	<0.001	1.118 (1.052–1.188)	<0.001
SCC	0.814 (0.556–1.184)	0.283	-	-
Smoking, Yes vs. No	3.08 (1.824–5.307)	<0.001	3.079 (1.716–5.526)	<0.001
TLG_d	1.008 (1.004–1.013)	<0.001	1.006 (1.001–1.011)	0.014
TLG_e	1.001 (0.998–1.005)	0.360	-	-
*Δ*TLG	1.483 (1.166–1.959)	0.003	1.549 (1.105–2.170)	0.011
SUVmax_e	1.03 (0.976–1.088)	0.294	-	-
SUVmax_d	1.064 (1.01–1.123)	0.022	1.070 (1.004–1.139)	0.036
*Δ*SUVmax	1.062 (1.031–1.1)	<0.001	1.046 (1.014–1.080)	0.005
MTV_e	0.995 (0.983–1.008)	0.482	-	-
MTV_d	1.015 (1.009–1.02)	<0.001	1.014 (1.006–1.023)	<0.001
*Δ*MTV	1.377 (1.181–1.628)	<0.001	1.078 (0.934–1.244)	0.304

OR, odds ratio; Cl, confidence interval; SCC, squamous cell carcinoma antigen; CEA, carcinoembryonic antigen; NSE, neuron-specific enolase; ProGRP, Pro-gastrin-releasing peptide; TLG_d, delayed phase total lesion glycolysis; TLG_e, early phase total lesion glycolysis; *Δ*TLG, percentage change in TLG; SUVmax_e, early phase maximum standardized uptake value; SUVmax_d, delayed phase maximum SUV; *Δ*SUVmax, percentage change in SUVmax; MTV_e, early phase metabolic tumor volume; MTV_d, delayed phase MTV; *Δ*MTV, percentage change in MTV.

### 3.4 Evaluation of predictive performance, robustness, and clinical utility of the XGBoost integrated model

This section systematically evaluates the discriminative performance of single-modality models and multimodal integrated models, screens the optimal model, and completes comprehensive validation across three dimensions: generalization robustness, predictive calibration, and clinical application value.

#### 3.4.1 Comparison of predictive performance between single-modality and integrated models

**Figure 3:**
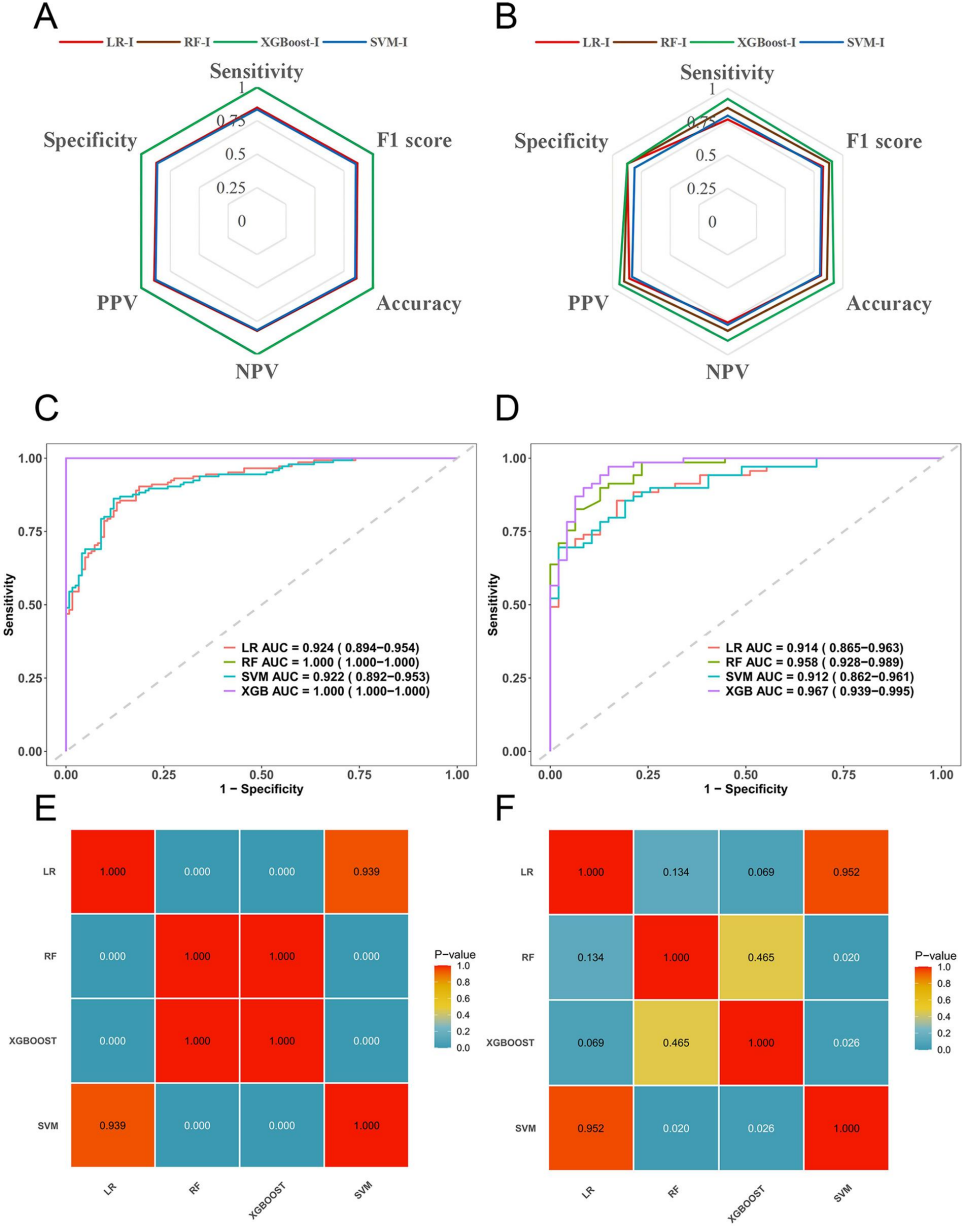
Comprehensive evaluation and comparison of machine learning model classification performance. **(A, B)**: Radar chart visualizations of predictive performance for diverse machine learning algorithms across the training set and independent temporal validation set. These multi-dimensional radar plots assess model performance through key metrics: Sensitivity, Specificity, Positive Predictive Value (PPV), Negative Predictive Value (NPV), and F1 Score (harmonic mean of Precision and Recall). The polygonal areas demonstrate trade-offs between metrics, highlighting algorithmic strengths and limitations in multi-criteria evaluation. **(C, D)**: Receiver operating characteristic (ROC) curves with corresponding Area Under the ROC Curve (AUC) values for the training set and independent temporal validation set. AUC, ranging from 0 to 1, quantifies classification capability with values closer to 1 indicating superior discriminatory power. Notably, Extreme Gradient Boosting (XGBoost) achieves near-perfect AUC scores (∼1.000) in both sets, underscoring its robust performance. **(E, F)**: AUC comparison matrix illustrating pairwise statistical significance via color-coded heatmaps. *P*-values (<0.05 denote statistical significance) reveal XGBoost's dominant performance across datasets, with significant AUC differences versus Logistic Regression (LR) and Support Vector Machine (SVM) (*p* < 0.05). Matrix diagonals represent self-comparisons (AUC=1.000), while off-diagonal cells quantify inter-model disparities.

Performance metrics for all models in the training set and independent temporal validation set are summarized in Table 3 and Figure 3. In the training set, the area under the receiver operating characteristic curve (AUC) was 0.813 for the Radiomics model, 0.761 for the Clinical model, and 0.816 for the Metabolic model. Among the four multimodal integrated models, the Logistic Regression integrated model (LR-I), Random Forest integrated model (RF-I), Support Vector Machine integrated model (SVM-I), and XGBoost integrated model (XGBoost-I) achieved AUCs of 0.924, 1.000, 0.922, and 1.000, respectively.

**Table 3:** Prediction performance of different models.

Cohort	Model	AUC (95% CI)	Accuracy	F1 score	Sensitivity	Specificity
Training	Radiomics model	0.813 (0.764–0.868)	0.772	0.810	0.897	0.626
	Clinical model	0.761 (0.704–0.818)	0.702	0.675	0.572	0.854
	Metabolic model	0.816 (0.766–0.867)	0.750	0.751	0.697	0.813
	LR-I	0.924 (0.894–0.954)	0.858	0.866	0.848	0.870
	RF-I	1.000 (1.000–1.000)	1.000	1.000	1.000	1.000
	XGBoost-I	1.000 (1.000–1.000)	1.000	1.000	1.000	1.000
	SVM-I	0.922 (0.892–0.953)	0.847	0.852	0.834	0.862
Validation	Radiomics model	0.808 (0.728–0.888)	0.759	0.806	0.841	0.638
	Clinical model	0.732 (0.641–0.823)	0.664	0.661	0.551	0.830
	Metabolic model	0.874 (0.811–0.936)	0.785	0.790	0.681	0.936
	LR-I	0.914 (0.865–0.963)	0.810	0.828	0.768	0.872
	RF-I	0.958 (0.928–0.989)	0.862	0.881	0.855	0.872
	XGBoost-I	0.967 (0.939–0.995)	0.921	0.905	0.923	0.872
	SVM-I	0.912 (0.862–0.961)	0.802	0.815	0.797	0.809

-I, integrated model; AUC, area under the curve; 95% CI, 95% confidence interval; LR, logistic regression; RF, random forest; XGBoost, eXtreme gradient boosting; SVM, support vector machine.

In the independent temporal validation set, the SVM-I model yielded an AUC of 0.902 (95% CI 0.856–0.948), an accuracy of 0.853, a sensitivity of 0.862, and a specificity of 0.830. The XGBoost-I model demonstrated the best overall performance, with an AUC of 0.967 (95% CI 0.939–0.995), a Brier Score of 0.0749 (95% CI 0.0462–0.1126), an accuracy of 0.921, an F1-score of 0.905, a sensitivity of 0.923, and a specificity of 0.872. DeLong's test performed on the temporal validation set confirmed that the XGBoost-I model had significantly better discriminative performance than the three single-modality models and the LR-I and SVM-I integrated models, with *P* values ranging from 0.002 to 0.032 (all *P* < 0.05).

Compared with the Radiomics model in the temporal validation set, the XGBoost-I model achieved a net reclassification improvement (NRI) of 0.321 (95% CI: 0.161–0.481, *P* < 0.001) and an integrated discrimination improvement (IDI) of 0.438 (95% CI: 0.351–0.526, *P* < 0.001), indicating that the XGBoost-I model provides a significant improvement in the individual risk stratification of malignant pulmonary lesions.

#### 3.4.2 Internal cross-validation and robustness assessment

To avoid data leakage and rigorously evaluate the model's generalizability and robustness, 5-fold cross-validation (for hyperparameter tuning) and 5 × 5 nested cross-validation (for unbiased robustness assessment) were performed exclusively within the training set. For all cross-validation procedures, hyperparameter tuning and model fitting were performed only in the training folds, with the validation folds completely isolated to ensure unbiased performance evaluation.

The mean ± standard deviation (SD) of performance metrics for the optimal XGBoost-I model in cross-validation are as follows:

5-fold cross-validation: AUC 0.958 ± 0.021, Brier Score 0.0812 ± 0.0125, accuracy 0.902 ± 0.034, sensitivity 0.915 ± 0.041, specificity 0.886 ± 0.038, F1-score 0.898 ± 0.036.

5 × 5 nested cross-validation: AUC 0.952 ± 0.028, Brier Score 0.0857 ± 0.0142, accuracy 0.897 ± 0.037, sensitivity 0.908 ± 0.045, specificity 0.882 ± 0.042, F1-score 0.893 ± 0.039.

The XGBoost-I model achieved an AUC of 1.000 in the full training set, and an AUC of 0.967 with a Brier Score of 0.0749 in the independent temporal validation set. DeLong's test showed no statistically significant difference in AUC between the full training set and the independent temporal validation set (*P* = 0.182).

#### 3.4.3 Calibration performance of the XGBoost-I model

**Figure 4:**
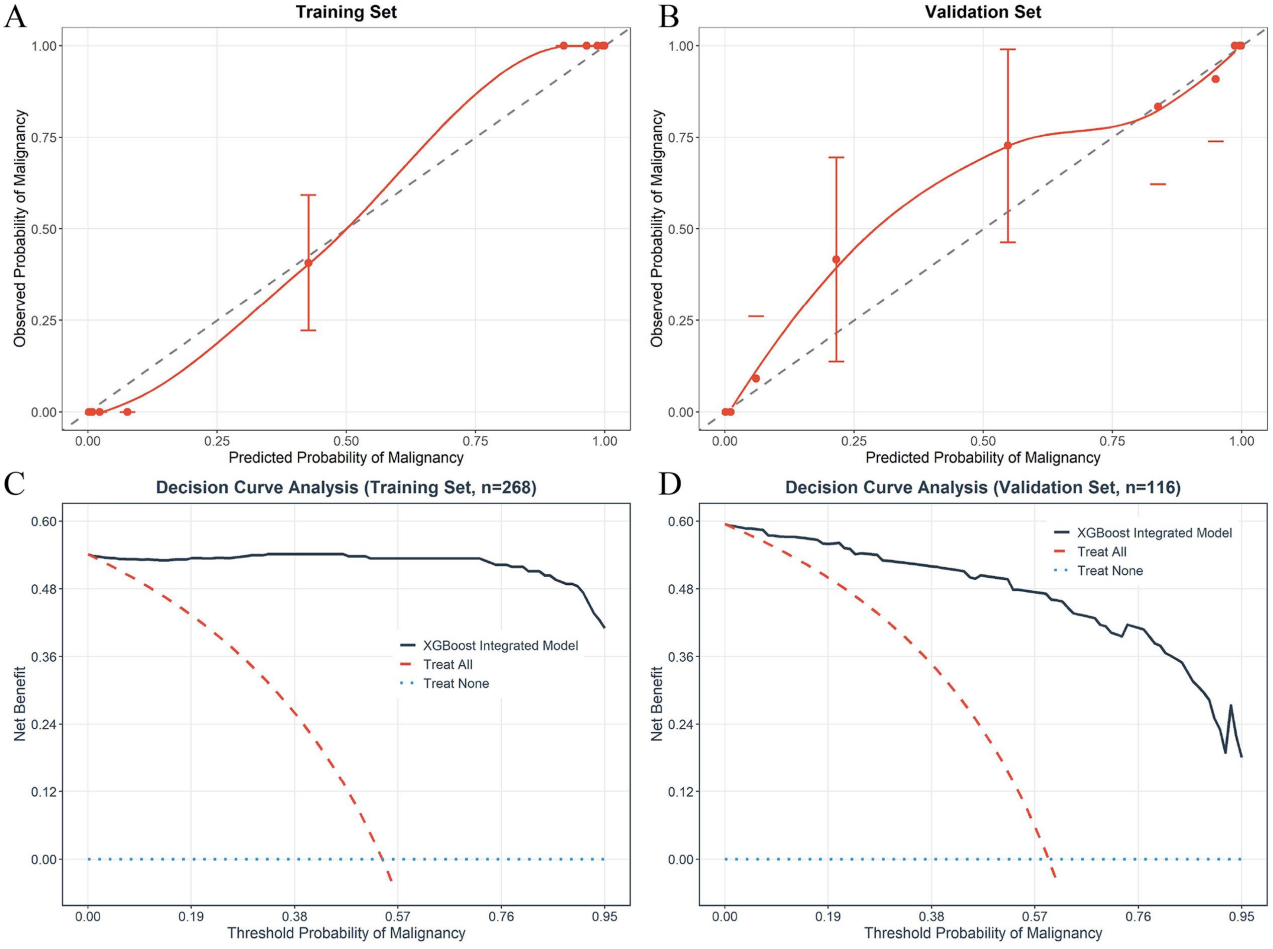
Calibration curves and decision curve analysis (DCA) of the XGBoost-I integrated model in the training set and independent temporal validation set. **(A)** Calibration curve in the training set; **(B)** Calibration curve in the independent temporal validation set. The diagonal line represents the ideal calibration, where the predicted probability of malignancy is perfectly consistent with the actual probability; the closer the model curve is to the diagonal, the better the calibration performance of the model. **(C)** DCA in the training set (*n* = 268); **(D)** DCA in the independent temporal validation set (*n* = 116). The *y*-axis represents the net benefit of the model, and the *x*-axis represents the threshold probability of malignancy. The XGBoost-I integrated model curve is compared with the two reference lines of “Treat All” (treating all lesions as malignant) and “Treat None” (treating all lesions as benign); a higher net benefit of the model curve over the reference lines indicates better clinical utility.

The calibration curves of the XGBoost-I model in the training set and temporal validation set are shown in Figures 4A, B, respectively. The curves were closely aligned with the ideal 45° diagonal line in both cohorts, indicating excellent agreement between the model-predicted malignancy probability and the actual observed risk of malignancy. The Hosmer-Lemeshow test further confirmed no statistically significant calibration deviation in either cohort (training set: *χ*^2^ = 6.898, df = 8, *P* = 0.548; temporal validation set: *χ*^2^ = 5.231, df = 8, *P* = 0.733). The excellent calibration performance ensures that the model's predicted probability is clinically reliable and can provide a solid reference for individualized clinical decision-making.

#### 3.4.4 Clinical utility assessment via decision curve analysis (DCA)

The DCA curves of the XGBoost-I model in the training set and temporal validation set are shown in Figures 4C, D, respectively. Within the clinically relevant threshold probability range of 0.1 to 0.8 (the core range for clinical decision-making of pulmonary lesion biopsy), the XGBoost-I model provided a significantly higher net benefit than the “treat all patients” and “treat none of the patients” strategies in both cohorts. Specifically, in the temporal validation set, the model achieved a net benefit of 0.40 to 0.55 within this threshold range, meaning that the model can reduce 40%–55% of unnecessary invasive biopsies for benign lesions without increasing the risk of missed diagnosis of malignant lesions. This result confirms that the XGBoost-I model has clear clinical application value and can effectively assist clinicians in individualized decision-making for the differential diagnosis of pulmonary space-occupying lesions.

### 3.5 SHAP interpretability analysis

**Figure 5:**
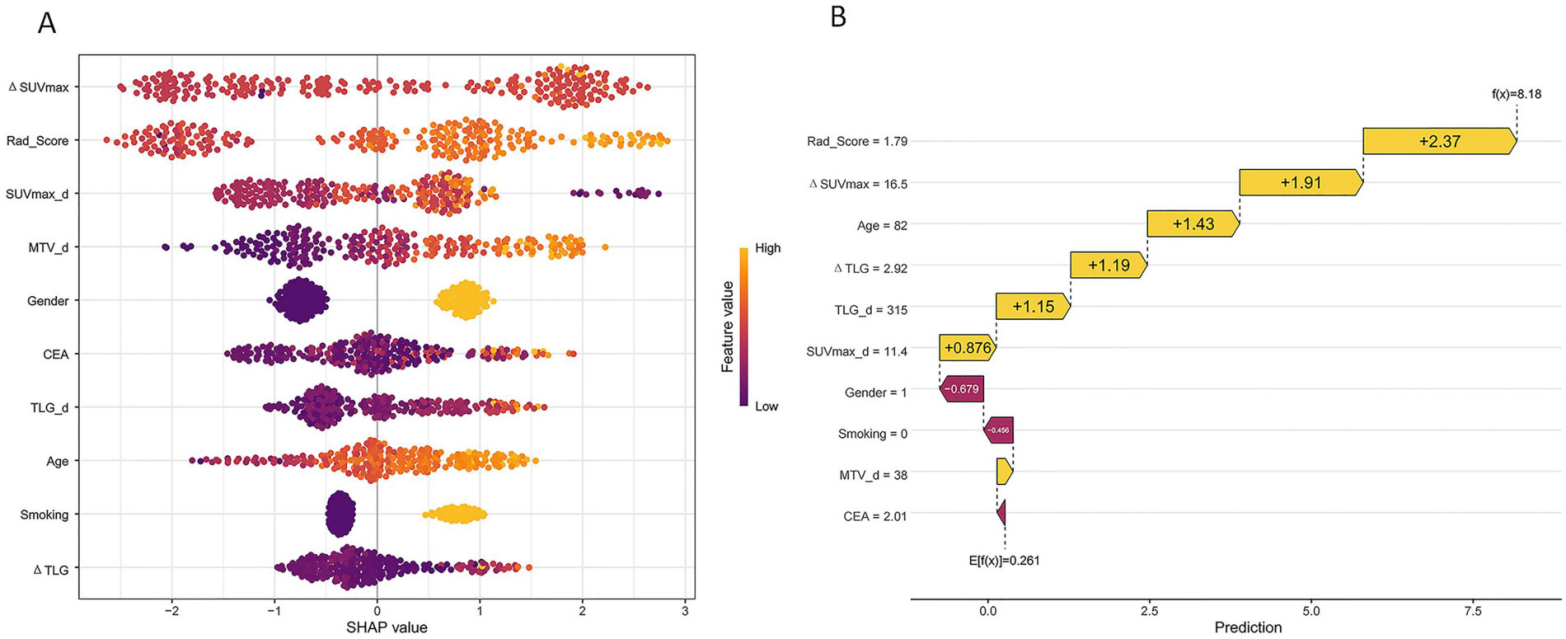
SHAP interpretability analysis of the optimal XGBoost integrated model. **(A)** SHAP beeswarm plot, showing the global feature importance ranking (sorted by mean absolute SHAP value from top to bottom) and the impact of each feature's value on the model's prediction. The *y*-axis represents the input features, with a higher position indicating a greater contribution to the model's benign-malignant differentiation. The *x*-axis represents the SHAP value: positive values increase the model's predicted probability of malignant PSOLs, while negative values increase the predicted probability of benign lesions. The color of each point represents the feature value from low (purple) to high (yellow). **(B)** SHAP waterfall plot, showing the contribution of each feature to the final prediction result of a single sample, explaining the model's decision logic at the individual patient level. The *x*-axis represents the model's predicted value, and the positive/negative values of each bar represent the direction of the feature's impact on the prediction result. *Δ*SUVmax, percentage change in maximum standardized uptake value; Rad-score, Radiomics Score; SUVmax_d, delayed phase maximum standardized uptake value; CEA, carcinoembryonic antigen; TLG_d, delayed phase total lesion glycolysis; *Δ*TLG, percentage change in total lesion glycolysis.

SHapley Additive exPlanations (SHAP) analysis was performed to decode the internal decision logic of the optimal XGBoost integrated model (the model with the best performance in the temporal validation set), quantify the relative contribution of each input feature to the model’s prediction results, and improve the transparency of the “black box” machine learning model (Figure 5).

Notably, the input feature set for SHAP analysis was completely consistent with the full feature set used for the final XGBoost model training, and all analysis was performed strictly based on the model trained exclusively within the training set, fully complying with the no-data-leakage principle.

The SHAP beeswarm plot showed the global feature importance ranking and the impact of each feature's value on the model's prediction (Figure 5A). *Δ*SUVmax was the feature with the highest contribution to the model's overall prediction, serving as the core driving factor for the model's benign-malignant differentiation of PSOLs, followed by Rad-score (the second most important feature) and SUVmax_d (the third most important feature). The subsequent ranking of feature importance was MTV_d, Gender, CEA, TLG_d, Age, Smoking history, and *Δ*TLG in turn. Notably, the Rad-score, as the second most critical predictive feature, has been validated to have excellent inter-observer and intra-observer reproducibility via strict ICC testing, ensuring the stability and reliability of the model's core input.

At the individual feature level, higher values of *Δ*SUVmax, Rad-score, SUVmax_d, MTV_d, CEA, TLG_d, Age, and *Δ*TLG were positively correlated with higher SHAP values, meaning they increased the model's predicted probability of malignant PSOLs; while Gender, non-smoking history were associated with lower SHAP values, meaning they increased the model's predicted probability of benign lesions (Figure 5A). The feature contribution pattern of the model was highly consistent with the independent predictive factors identified by multivariable Logistic regression, and aligned with the clinical diagnostic routine of PSOLs, further verifying the rationality of the model's feature selection.

In addition, the SHAP waterfall plot was used to explain the model’s decision logic at the individual patient level, showing the contribution of each feature to the final prediction result of a single sample, which further improved the clinical interpretability of the model’s decision-making process (Figure 5B).

### 3.6 Nomogram

**Figure 6:**
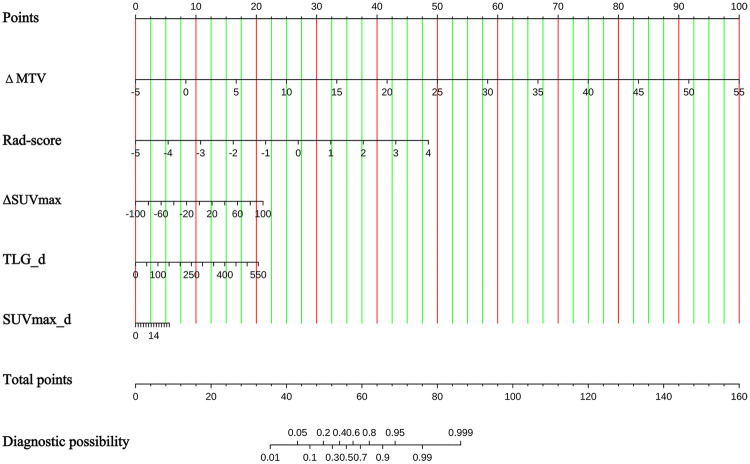
Nomogram for predicting the malignancy risk of pulmonary space-occupying lesions. *Δ*MTV, percentage change in metabolic tumor volume; Rad-score, radiomics score; *Δ*SUVmax, percentage change in maximum standardized uptake value; TLG_d, delayed phase total lesion glycolysis; SUVmax_d, delayed phase maximum SUV.

To facilitate the clinical translation of our optimal prediction model, a visual nomogram was developed based on the core predictive features identified by SHAP analysis of the XGBoost integrated model (Figure 6). The nomogram incorporates 5 key features with the highest predictive contribution, including Rad-score, *Δ*SUVmax, TLG_d, SUVmax_d, and *Δ*MTV, all of which were confirmed to have non-negligible global importance for benign-malignant differentiation of PSOLs via SHAP interpretability analysis. A multivariate logistic regression model was constructed using these 5 core features, and the nomogram was further generated based on this linear model.

In clinical practice, clinicians can locate the patient's measured value for each feature on the corresponding axis, sum the points assigned for each feature to get a total score, and then project the total score onto the ‘Risk of Malignancy’ axis to obtain the individualized predicted probability of a malignant lesion. This nomogram simplifies the complex prediction process of the machine learning model into an intuitive, easy-to-use scoring tool, while retaining the core predictive performance of the optimal XGBoost model, serving as a practical auxiliary tool for clinical decision-making.

## 4 Discussions

This study integrated CT radiomics, dual-time-point PET metabolic parameters, and clinical data to develop and validate a high-performance multi-modal machine learning model for the benign-malignant differentiation of pulmonary space-occupying lesions (PSOLs). Through systematic comparison of four mainstream machine learning algorithms, we confirmed that the XGBoost integrated model (XGBoost-I) achieved the best discriminative performance, with an AUC of 0.967 in the independent temporal validation set. We further decoded the “black-box” model via SHAP interpretability analysis, and developed a clinically applicable nomogram based on the core predictive features, establishing a complete workflow from high-accuracy model construction to clinical translation.

In terms of model performance, the XGBoost-I model achieved an AUC of 0.967 (95% CI: 0.939–0.995), accuracy of 0.921, F1-score of 0.905, sensitivity of 0.923, and specificity of 87.2% in the independent temporal validation set, which significantly outperformed all single-modality models (radiomics model AUC=0.808, clinical model AUC=0.732, metabolic model AUC=0.874). Compared with previous similar studies, our model showed a clear performance advantage: Kang et al. (9) reported an AUC of 0.89 for a PET/CT radiomics model in PSOL differentiation, while our multi-modal integrated model further improved the AUC to 0.967, which is mainly attributed to the comprehensive fusion of three data modalities (CT radiomics, dual-time-point PET metabolic parameters, and clinical data) and the nonlinear fitting capability of the XGBoost algorithm. This finding is consistent with the study by Meng et al. (14), which confirmed the superiority of the XGBoost algorithm in complex medical prediction scenarios with multi-source heterogeneous data.

SHAP interpretability analysis further decoded the internal decision logic of the optimal XGBoost-I model, and clarified the relative contribution of each feature to benign-malignant differentiation, strictly consistent with the global feature importance ranking in Figure 5A. *Δ*SUVmax was the most important predictive feature (ranked first): higher *Δ*SUVmax values were significantly associated with an increased predicted probability of malignancy, which is fully consistent with established clinical evidence that *Δ*SUVmax from dual-time-point PET imaging reflects the dynamic glucose metabolic activity of lesions, a core hallmark of malignant tumors (15, 16). Rad-score ranked as the second most critical feature: it showed an extremely wide distribution of SHAP values in both the training set (−4.97 to 3.25) and the independent temporal validation set (−2.65 to 2.75), with significantly higher values in the malignant group than in the benign group (*P* < 0.001). The Rad-score was constructed from 17 stable radiomic features (82.6% textural features), which can quantitatively characterize the intratumoral heterogeneity of PSOLs, including irregular borders, internal density variations, and spatial pixel distribution patterns that are typical of malignancy (17, 18). SUVmax_d ranked third, which directly reflects the maximum glucose metabolic activity of the lesion in the delayed phase, and also exhibited strong predictive power for malignant differentiation. In addition, TLG_d, as another important metabolic parameter with non-negligible predictive contribution, exhibited high absolute SHAP values; by integrating overall metabolic activity and tumor volume, it provides a comprehensive measure of the total metabolic burden of the tumor, thus also exerting a significant impact on malignancy prediction (19).

Notably, the use of dual-time-point PET imaging is a key factor supporting the high performance of our model. Compared with single-time-point PET, it provides richer dynamic metabolic information through parameters such as *Δ*SUVmax and *Δ*TLG, capturing the changes in glucose uptake between early and delayed scans. Malignant tumors often show continuously increasing metabolic activity on delayed imaging, while benign lesions usually exhibit stable or decreasing uptake (20). SHAP analysis further confirmed the clinical relevance of these dynamic parameters, which effectively enhanced the model's ability to distinguish between malignant lesions and inflammatory benign lesions with high static metabolic uptake, thus reducing the false positive rate of traditional PET/CT diagnosis.

Our study has three core methodological advantages that address the key limitations of existing radiomics studies on PSOLs. First, we adopted a strict temporal split strategy at a 7:3 ratio for the training and validation sets, with all feature selection, hyperparameter tuning, and model training performed exclusively within the training set, which completely avoided data leakage and rigorously verified the model's generalizability on completely unseen data. Second, we overcame the limitations of traditional linear statistical methods: the XGBoost algorithm, as a nonlinear tree-based ensemble model, can effectively capture the nonlinear predictive value of features and the synergistic interaction between multiple features, even if the feature does not show independent linear statistical significance in traditional logistic regression. Notably, the XGBoost-I model achieved a perfect AUC of 1.000 in the full training set, while maintaining an excellent AUC of 0.967 in the independent temporal validation set, with no statistically significant performance degradation confirmed by DeLong's test (*P* = 0.182). The internal 5-fold and 5 × 5 nested cross-validation results (AUC ∼0.95) were highly consistent with the validation set performance, and the Brier score of the model was below the 0.1 threshold for excellent predictive accuracy in both cohorts, collectively confirming that the model has no severe overfitting that would affect clinical generalization. Third, we established a complete workflow for the clinical translation of the machine learning model: we first maximized the predictive performance through the nonlinear XGBoost algorithm, then decoded the “black-box” model via SHAP analysis to screen the core predictive feature set, and finally developed an intuitive, clinically operable nomogram based on a multivariate logistic regression model using these core features. This workflow not only ensures the high accuracy of the prediction model, but also solves the clinical application limitation of the machine learning “black box” (21).

The calibration and decision curve analysis results further validated the clinical translational value of our model. The model showed excellent calibration in both the training and validation cohorts, with calibration curves closely aligned to the ideal 45° diagonal and no significant deviation confirmed by the Hosmer-Lemeshow test (training set *P* = 0.548; validation set *P* = 0.733), ensuring the reliability of the predicted malignancy probability. Meanwhile, DCA confirmed that the model provided a significantly higher clinical net benefit than the “treat all” and “treat none” strategies across the clinically relevant threshold range of 0.1 to 0.8, which means it can reduce 40%–55% of unnecessary invasive biopsies for benign lesions without increasing the risk of missed diagnosis of malignant lesions. The nomogram we developed further simplifies the complex prediction process of the machine learning model into an easy-to-use clinical tool: it incorporates 5 core predictive features (Rad-score, *Δ*SUVmax, SUVmax_d, TLG_d, and *Δ*MTV) identified by SHAP analysis, and clinicians can quickly calculate the individualized malignancy risk of a patient through a simple scoring process (22, 23). This nomogram retains the core predictive performance of the optimal XGBoost-I model, while providing clear interpretability and operability for clinical decision-making (24).

Despite the encouraging performance and clinical translational potential of our model, several limitations should be acknowledged. First, this single-center retrospective study lacks independent multi-center external validation, the core limitation of this work. Although strict temporal validation and standardized single-scanner imaging protocols were adopted to eliminate intra-study batch effects, the model's performance to cohorts with different scanners, protocols, and patient populations remains to be verified. We will conduct multi-center external validation with standardized imaging harmonization strategies in future work. Second, the retrospective design may introduce selection bias, and the sample size may insufficiently capture the heterogeneity of rare PSOL subtypes. Meanwhile, manual segmentation, despite excellent reproducibility, is labor-intensive for large-scale application; we will explore automated segmentation algorithms in larger multi-center cohorts to optimize the workflow. Third, the biological mechanisms underlying the selected radiomic features require further radiogenomic validation. Additionally, SHAP analysis can only explain the model's decision logic and feature contributions, but cannot infer direct biological causality between features and PSOL malignant behavior, which warrants further pathological and radiogenomic verification. Finally, we did not perform systematic pathological subtype stratification for benign and malignant PSOLs. Given the significant radiomic and metabolic heterogeneity across different pathological subtypes, we will conduct subtype-specific subanalysis in the subsequent multi-center study to further optimize the model's robustness and diagnostic performance.

## 5 Conclusions

This study developed and validated an interpretable multi-modal machine learning model integrating CT radiomics, dual-time-point PET metabolic parameters, and clinical data for the benign-malignant differentiation of PSOLs. The model shows excellent discriminative performance, calibration, and clinical utility, and the nomogram we established provides a practical, easy-to-use tool for individualized risk stratification of patients with PSOLs, which has important clinical application value.

## Data Availability

The original contributions presented in the study are included in the article/Supplementary Material, further inquiries can be directed to the corresponding author.
