# Supplementary material for: Development and validation of a nomogram using interpretable machine learning to integrate CT radiomics and PET metabolic parameters for predicting benign-malignant differentiation of pulmonary space-occupying lesions
Source: Front Radiol. 2026 Apr 21;6:1782678. doi: 10.3389/fradi.2026.1782678 (PMC13139110; doi:10.3389/fradi.2026.1782678)
Supplement: Supplementary file 1 [file Table1.docx]

**Supplementary Table S1. Full details of the 17 core radiomic features selected by LASSO regression**

| **Feature Full Name** | **Feature Category** | **LASSO Coefficient** | **ICC Value** | **P value**  **(Malignant vs Benign)** |
| --- | --- | --- | --- | --- |
| original_shape_Flatness | Shape feature | 0.7563 | 0.924 | <0.001 |
| original_shape_MajorAxisLength | Shape feature | 0.0056 | 0.957 | <0.001 |
| original_shape_Sphericity | Shape feature | 2.3994 | 0.891 | <0.001 |
| original_shape_VoxelVolume | Shape feature | 8.96×10⁻⁶ | 0.962 | <0.001 |
| original_firstorder_10Percentile | First-order feature | 0.0010 | 0.876 | <0.001 |
| original_firstorder_90Percentile | First-order feature | 0.000367 | 0.883 | <0.001 |
| original_glcm_InverseVariance | Original GLCM feature | 2.8237 | 0.852 | <0.001 |
| original_gldm_SmallDependenceHighGrayLevelEmphasis | Original GLDM feature | 0.0025 | 0.786 | <0.001 |
| original_glrlm_RunLengthNonUniformity | Original GLRLM feature | -5.7×10⁻⁶ | 0.814 | <0.001 |
| original_glszm_GrayLevelNonUniformityNormalized | Original GLSZM feature | 0.7458 | 0.795 | <0.001 |
| wavelet.LLH_firstorder_Kurtosis | Wavelet-transformed first-order feature | -0.0138 | 0.837 | <0.001 |
| wavelet.LLH_firstorder_Mean | Wavelet-transformed first-order feature | 0.0089 | 0.902 | <0.001 |
| wavelet.LLH_glcm_ClusterProminence | Wavelet-transformed GLCM feature | 1.6×10⁻⁶ | 0.865 | <0.001 |
| wavelet.LLH_glcm_Idmn | Wavelet-transformed GLCM feature | 31.9905 | 0.889 | <0.001 |
| wavelet.LLH_glcm_MCC | Wavelet-transformed GLCM feature | 5.3096 | 0.913 | <0.001 |
| wavelet.LLH_gldm_DependenceVariance | Wavelet-transformed GLDM feature | 0.0080 | 0.778 | <0.001 |
| wavelet.LLH_glrlm_ShortRunLowGrayLevelEmphasis | Wavelet-transformed GLRLM feature | -4.5512 | 0.763 | <0.001 |
